# Supplementary material for: Reconciling Mining with the Conservation of Cave Biodiversity: A Quantitative Baseline to Help Establish Conservation Priorities
Source: PLoS One. 2016 Dec 20;11(12):e0168348. doi: 10.1371/journal.pone.0168348 (PMC5173368; doi:10.1371/journal.pone.0168348)
Supplement: S1 Dataset — (ZIP) [file pone.0168348.s002.zip › Taxa/Serra Norte/SN_2013/N5S/Lista N5S-59.pdf]

## CAVIDADE N5S-0059

| Classe | Ordem | Fam/Outros | Gên/Outros | Espécie | S-U | U-S | Zon. |
|---|---|---|---|---|---|---|---|
| Arachnida | Acari | Anystidae | *Erythracarus* | sp.1 | X | | E |
| Arachnida | Amblypygi | Phrynidae | *Heterophrynus* | *longicornis* | 1 | 1 | E/P |
| Arachnida | Araneae | Araneidae | *Alpaida* | aff. *negro* | | X | E |
| Arachnida | Araneae | Filistatidae | | sp.1 | X | X | E/P |
| Arachnida | Araneae | Gnaphosidae | | jovem | | X | E |
| Arachnida | Araneae | Mysmenidae | *Microdipoena* | sp.1 | X | | E |
| Arachnida | Araneae | Oonopidae | *Gamasomorpha* | sp.1 | X | X | P |
| Arachnida | Araneae | Pholcidae | *Leptopholcus* | sp.1 | | X | P |
| Arachnida | Araneae | Pholcidae | *Mesabolivar* | *aurantiacus* | | X | E/P |
| Arachnida | Araneae | Pholcidae | | jovem | X | | E |
| Arachnida | Araneae | Salticidae | | jovem | X | | E/P |
| Arachnida | Araneae | Segestriidae | | jovem | X | | P |
| Arachnida | Araneae | Tetragnathidae | *Leucauge* | sp.1 | | X | E |
| Arachnida | Araneae | Theraphosidae | | jovem | | 1 | E |
| Arachnida | Araneae | Theridiidae | *Theridion* | sp.1 | X | X | P |
| Arachnida | Araneae | Theridiosomatidae | *Plato* | sp.1 | X | X | P |
| Arachnida | Opiliones | Sclerosomatidae | *Prionostema* | sp.1 | | 1 | E |
| Insecta | Blattodea | Blattellidae | | sp.1 | 5 | 7 | E/P |
| Insecta | Blattodea | Polyphagidae | | sp.1 | | 2 | P |
| Insecta | Coleoptera | Coccinellidae | | sp.2 | X | X | E |
| Insecta | Diptera | Cecidomyiidae | Cecidomyiinae | sp. | X | X | E |
| Insecta | Diptera | Culicidae | *Culex* | sp. | X | | E |
| Insecta | Diptera | Dolichopodidae | | sp. | X | | E/P |
| Insecta | Diptera | Ephydridae | | sp. | | X | E/P |
| Insecta | Diptera | Psychodidae | Phlebotominae | sp. | X | X | E/P |
| Insecta | Diptera | Tipulidae | Tipulinae | sp. | | X | E/P |
| Insecta | Heteroptera | Dipsocoroidea | | jovem | X | | P |
| Insecta | Heteroptera | Reduviidae | Reduviinae | sp. | 2 | | E |
| Insecta | Hymenoptera | Braconidae | | sp.2 | X | | E |
| Insecta | Hymenoptera | Formicidae | *Camponotus* | sp.2 | X | X | P |
| Insecta | Hymenoptera | Formicidae | *Crematogaster* | sp.1 | X | X | E/P |
| Insecta | Hymenoptera | Formicidae | *Gnamptogenys* | sp.1 | | X | E |
| Insecta | Hymenoptera | Formicidae | *Pachycondyla* | sp.2 | X | | P |
| Insecta | Isoptera | Termitidae | *Nasutitermes* | sp. | X | X | E/P |
| Insecta | Lepidoptera | Gelechioidea | | sp. | | X | E/P |
| Insecta | Lepidoptera | Noctuoidea | | sp.5 | 1 | | P |
| Insecta | Lepidoptera | Tineoidea | | sp.3 | X | X | E/P |
| Insecta | Orthoptera | Phalangopsidae | *Paraclodes* | sp. | 3 | | E/P |
| Insecta | Orthoptera | Phalangopsidae | *Phalangopsis* | sp. | 18 | 5 | E/P |
| Insecta | Psocoptera | Epipsocidae | *Epipsocus* | sp.1 | | X | P |
| Insecta | Psocoptera | Psyllipsocidae | *Psyllipsocus* | sp.1 | X | | E |
| Insecta | Psocoptera | Ptiloneuridae | *Ptiloneura* | sp.1 | X | | P |
| Insecta | Thysanura | Nicoletiidae | | jovem | X | | E |
| Malacostraca | Isopoda | Dubioniscidae | | sp.1 | X | X | E |
| Malacostraca | Isopoda | Philosciidae | | sp.2 | X | | E |
| Amphibia | Anura | | | sp. | 1 | | P |
| Mammalia | Chiroptera | Emballonuridae | *Peropteryx* | *kappleri* | 3 | | E/P |
| Mammalia | Chiroptera | Furipteridae | *Furipterus* | *horrens* | 2 | 5 | E/P |
| Mammalia | Rodentia | Cricetidae | *Rhipidomys* | sp. | | 1 | P |

| | | | | | | | |
|---|---|---|---|---|---|---|---|
| Reptilia | Squamata | Sauria | | sp. | | 2 | E/P |
